# At a crossroads: Genetic lineages and dispersal routes of *Morimusasper* (Sulzer, 1776) s.l. (Coleoptera, Cerambycidae) in Bulgaria

**DOI:** 10.3897/BDJ.12.e116619

**Published:** 2024-02-05

**Authors:** Rumyana Kostova, Simeon Borissov, Aneliya Bobeva, Rostislav Bekchiev

**Affiliations:** 1 Sofia University, Faculty of Biology, Sofia, Bulgaria Sofia University, Faculty of Biology Sofia Bulgaria; 2 Institute of Biodiversity and Ecosystem Research, Bulgarian Academy of Sciences, Sofia, Bulgaria Institute of Biodiversity and Ecosystem Research, Bulgarian Academy of Sciences Sofia Bulgaria; 3 National Museum of Natural History, Bulgarian Academy of Sciences, Sofia, Bulgaria National Museum of Natural History, Bulgarian Academy of Sciences Sofia Bulgaria

**Keywords:** longhorned beetles, Lamiinae, COI, ITS2, genetic diversity, Balkans

## Abstract

The present study fills a knowledge gap in the distribution and genetic variation of *Morimus* populations in the Balkans, by studiyng the representatives of the genus in Bulgaria – *M.asperfunereus* Mulsant, 1862, *M.verecundusbulgaricus* Danilevsky, 2016 and *M.orientalis* Reitter, 1894. Additional information is provided for Albania and northern Greece. The mitochondrial cytochrome C oxidase subunit I (COI) marker and the nuclear internal transcribed spacer 2 (ITS2) were used for the genetic analyses. Three of the previously-defined mitochondrial lineages (Lb/HgA, L2 and L3) were detected in Bulgaria, as well as a new lineage (Str) from the Strandzha Mountains (south-eastern Bulgaria). A total of 24 distinct haplotypes, 20 of them in Bulgaria, were found. Bulgarian populations of *Morimus* demonstrated relatively high nucleotide diversity. The L3 COI lineage was confirmed as the most diverse and frequent in the Balkans. The L3 lineage is dominant in most of Bulgaria, but was not identified in the easternmost parts near the Black Sea coast, where the L2 and Str lineages were found. New data highlighted two dispersal routes of the L2 mitochondrial lineage on the Balkan Peninsula: 1) northwards along the Black Sea coast and 2) westwards, across the Balkans where only disjunct populations remain. North-western Bulgaria seems to be the eastern limit of the basal lineage Lb/HgA distribution. Our results show high levels of genetic exchange between most of the mitochondrially defined lineages, yet some of the easternmost populations probably remained isolated for comparatively longer periods.

## Introduction

The members of the genus *Morimus* Brullé, 1832 are saproxylic longhorned beetles, widespread in central, southern and eastern Europe and in some regions of western Asia. They inhabit mostly old deciduous forests and possess a limited dispersal ability as they are flightless (Sama and Löbl 2010, Hardersen et al. 2017). The European and Oriental members of the genus show intra- and interpopulation variability and overlap in the features used for taxonomic identification (Solano et al. 2013). Solano et al. (2013), based on the cytochrome C oxidase subunit I (COI) and the internal transcribed spacer 2 (ITS2) sequences, reasonably stated that all European and Turkish populations of *Morimus* should be referred to *M.asper* (Sulzer, 1776) and all other taxa have infraspecific position. The study covered the territories of Italy, Slovenia, Croatia, Montenegro, Greece, Turkey and Iran, defining the four main phylogeographic mitochondrial lineages of the *M.asper* s.l.: a basal lineage (Lb) consisting of five haplogroups (HgA-E) and three others – L1, L2 and L3. Giannoulis et al. (2020) added information from France and central Greece and confirmed *M.asperasper* and *M.asperfunereus* as close forms by studying COI mutations. They also revealed similar karyotypes and behaviour of both subspecies and their ability to mate in captivity. Gojković et al. (2022) completed the data by investigating the genetic diversity of the species in Serbia, supporting their study with geometric morphometrics. They found 15 distinct haplotypes from the lineages Lb-HgA; L2 and L3 sensu Solano et al. (2013) on the territory of Serbia and defined at least two evolutionarily and demographically distinct groups within the *M.asper* complex in the Balkans. The ITS2 sequences indicate an entirely homogeneous population. The geometric morphometric data confirmed the obtained genetic data of the Serbian population of *Morimusasper* s.l. (Gojković et al. 2022).

According to the latest publication by Danilevsky (2020) and Tavakilian and Chevillotte (2024), based on morphological characteristics, at least three species of the genus *Morimus* are present in Bulgaria: *Morimusasperfunereus* Mulsant, 1862, *M.verecundusbulgaricus* Danilevsky, 2016 and *M.orientalis* Reitter, 1894. Other authors have treated *M.verecundus* (Faldermann, 1836) as a subspecies of *M.asper* (Sama and Löbl 2010). *Morimusasper* s.l. populations in Bulgaria are associated with beech forests and (to a much lesser extent) with oak and other deciduous forests, mainly in lowland forests (Kostova et al. 2023). *Morimusasperfunereus* is widespread throughout Bulgaria; *M.verecundusbulgaricus* has an extremely localised population – until now found only in the Botanical Gardens of Sofia University in Balchik and Varna (north Black Sea coast) and *M.orientalis* is with confirmed localities only in the Strandzha Mts. (Bringmann 1996, Danilevski et al. 2016, Georgiev et al. 2018).

Despite imperfections, due to different evolutionary processes that could bring discordance between gene and species trees (Mallo and Posada 2016, Gojković et al. 2022), COI mtDNA is the most widely used marker for species delimitation and barcoding to date (Hebert et al. 2003, Dietz et al. 2022). Understandably, COI provides the most complete and comparable picture of genetic variation in *Morimus*.

The present study aims to extend the knowledge on the phylogenetic lineages distribution and genetic diversity of *M.asper* s.l. by extensive sampling in Bulgaria and neighbouring countries and to outline some routes of dispersal out of Pleistocene refugia that have remained undiscussed.

## Material and methods

**Sampling, molecular procedures and data preparation**

**Figure 1.**
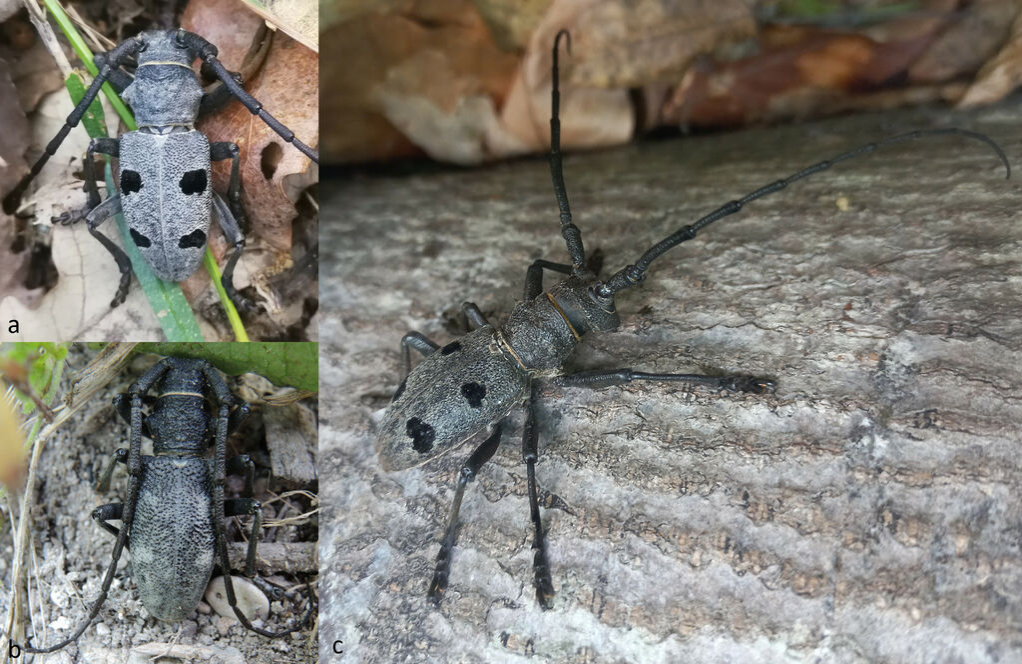
Habitus of the representatives of genus *Morimus* in Bulgaria. **a**) *M.asperfunereus* (Vitosha Mts., Bosnek vill.); **b**) *M.verecundusbulgaricus* (Balchik, Botanical Garden, © O. Sivilov); and **c**) *M.orientalis* (Strandzha Mts., Kosti vill.)

Material was collected between 2019 and 2022 from Bulgaria, Albania and Greece (northern part). Sampling was targeted to cover populations of *M.asperfunereus*, *M.verecundusbulgaricus* (type locality) and *M.orientalis* (Strandzha Mts.) (Fig. 1). A total of 62 samples (adult specimens or one of their hind legs) were collected and stored in absolute alcohol at -20°C. Total DNA was isolated from the hind femur by using a Qiagen DNeasy Blood & Tissue Kit following the instructions of the manufacturer. New data were obtained for two molecular markers: a fragment from the mitochondrial cytochrome C oxidase I gene (COI), amplified with primers C1-J-2183 and TL2-N-3014 (Simon et al. 1994) and a fragment from the nuclear internal transcribed spacer 2 (ITS2), amplified together with the 5.8S rRNA gene using primers TAGAGGAAGTAAAAGTCG forward and GCTTAAATTCAGCGG reverse (Weekers et al. 2001). Polymerase chain reaction (PCR) was carried out using HotStarTaq *Plus* Master Mix (Qiagen Inc). Thermal cycling followed Solano et al. (2013) for COI and Weekers et al. (2001) for ITS2. Sanger sequencing was carried out by Macrogen Europe BV.

Chromatograms were processed with CodonCode Aligner v.8.0.2 (CodonCode, Dedham, MA, USA). The obtained COI and ITS2 sequences were submitted to GenBank under accession numbers OR827907- OR827968 and OR844461- OR844487, respectively. Our own data were compiled with DNA sequences from previous studies (Solano et al. 2013, Gojković et al. 2022), accessed from GenBank (Suppl. material 1). Alignments were created using Mega X (Kumar et al. 2018). Protein-coding sequences were checked for stop codons with DAMBE v.7.0.39 (Xia 2018).

**Molecular phylogeny and demographic analyses**

The mitochondrial phylogenetic analysis was based on the COI fragment and the newly-obtained 62 sequences were compiled with the published data — 54 sequences (Solano et al. 2013, Gojković et al. 2022) (Suppl. material 1). The ITS2 analysis was based on 107 ingroup sequences from which 27 new and 80 published (Solano et al. 2013, Gojković et al. 2022) (Suppl. material 1). The taxon *Lamiatextor* (Linnaeus 1758), sequence MN886082 (Souza et al. 2020) was included as an outgroup for the COI phylogenetic analysis and the taxon *Iberodorcadionperezi* (Graëlls, 1849), sequence OK048710, was used as an outgroup for the ITS2 phylogenetic analysis (Dascălu et al. 2022).

The best substitution models (for each coding position of COI and for ITS2 as a single partition) were estimated with PartitionFinder ver. 2.1.1 (Lanfear et al. 2016). Phylogeny was reconstructed through Bayesian Inference (BI) accomplished in MrBayes v. 3.2.7 (Ronquist et al. 2012). The analysis used four simulations of Markov chains with 4 × 10^6^ generations, sampling 1 of every 100 trees. Chain parameters were checked for convergence in Tracer ver. 1.7.1 (Rambaut et al. 2018). The first 25% of trees were discarded as burn-in.

Nucleotide diversity, Tajima’s D (Tajima 1989), Fu and Li D' and F' test statistics (Fu and Li 1993) and Fu’s Fs (Fu 1997) were calculated in DnaSP v.6 (Rozas et al. 2017) to infer population dynamics. Confidence intervals were obtained through coalescent simulations under the Standard Neutral Model (SNM). Haplotype networks were created using the TCS algorithm (Clement et al. 2000) implemented in PopART 1.7 (Leigh and Bryant 2015). The mitochondrial haplotype network used our own and published COI sequences, while a 5.8S-ITS2 fragment was used for new data only (see below).

## Results

**Distribution of genetic diversity in the eastern Balkans**

- COI gene

**Figure 2.**
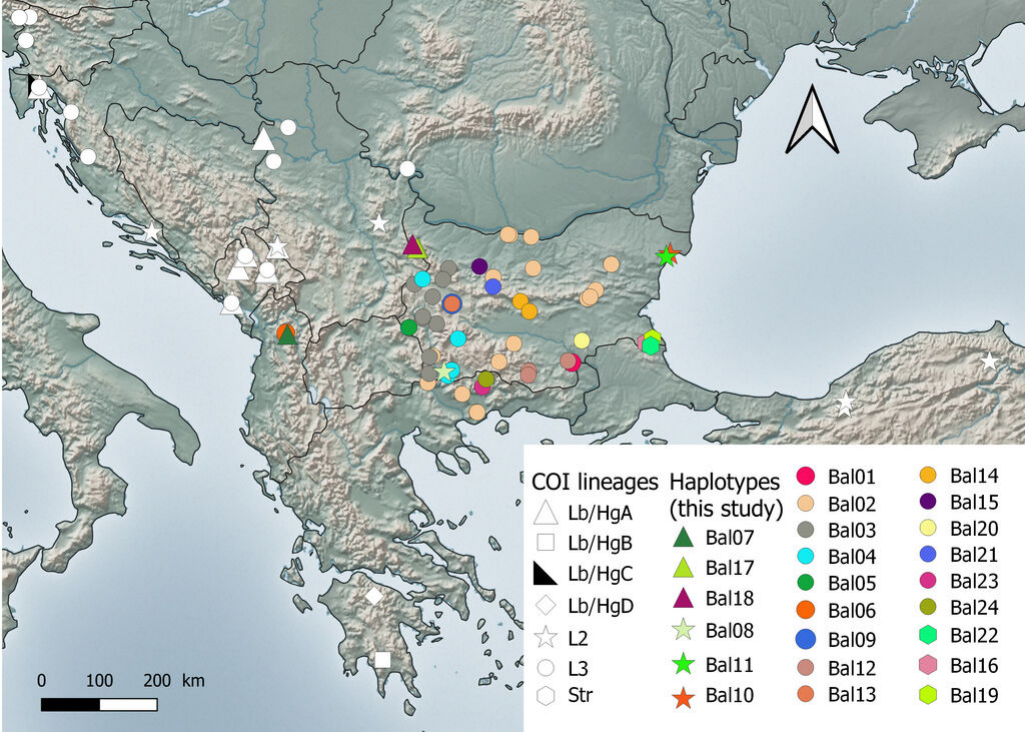
Distribution of the COI lineages of *Morimusasper* s.l. in the Balkans and neighbouring parts of Turkey (including data by Solano et al. (2013) and Gojković et al. (2022)) and the haplotypes obtained from the present study. Shape of the symbols indicates the COI lineages and the colour indicates the haplotypes.

The final length of the COI fragment was 756 bp. A total of 62 new *Morimusasper* s.l. COI sequences were obtained from Bulgaria, northern Greece and Albania. These represented 24 distinct haplotypes (Suppl. material 1). Three of the mitochondrial lineages discussed in Solano et al. (2013) were detected, as well as one distinct lineage from the Strandzha Mountains (southeast Bulgaria). More than 80% of the samples (15 distinct haplotypes) found across the whole territory of Bulgaria, northern Greece and northern Albania arranged within the L3 lineage. The basal lineage, haplogroup HgA (Lb-HgA) was found in the westernmost part of the Stara Planina Mts. close to the border with Serbia. The L2 lineage was found only in south-western (Melnik in the foothills of the Pirin Mts.) and in north-eastern Bulgaria (Baltata, Albena). One sample, morphologically identified as *M.verecundus* (Balchik), also arranges within the L2 lineage. Three unique haplotypes were found in the Strandzha Mountains showing 2−3% genetic distance from the L3 lineage. A detailed map of the distribution of COI lineages in the Balkans and north-western Turkey, as well as the haplotypes obtained in this study, is shown in Fig. 2.

- ITS gene

Overall, 27 new sequences from the nuclear 5.8S-ITS2 partial fragment were obtained in this study. The final length of the fragment was 373 bp, including gaps. Our own DNA sequences were aligned with those from Solano et al. (2013) and Gojković et al. (2022) for comparison. The majority of the new samples demonstrate the Mor ITS2 (1a) genotype. The sample from the Balchik Botanical Garden (morphologically *M.verecundus*) showed a unique sequence, although very similar to the Mor ITS2 (5a) haplotype from the Giresun Region in Turkey (Suppl. material 1, Suppl. material 3).

**Phylogeny and population dynamics**

**Figure 3.**
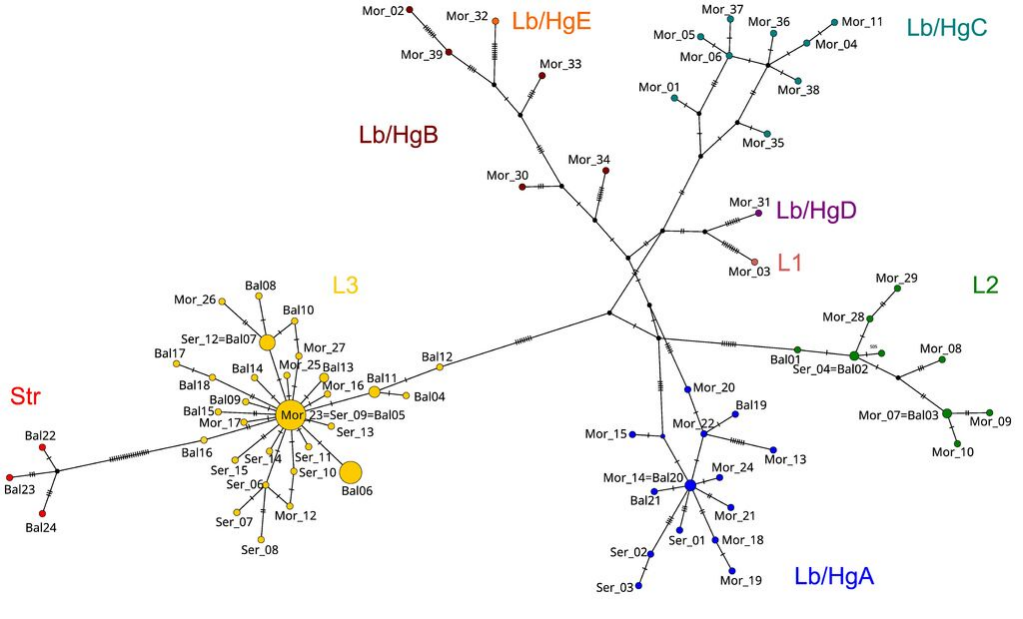
TCS network of the mitochondrial COI haplotypes of all samples. The original signatures of the haplotypes from Solano et al. (2013) and Gojković et al. (2022) are used and the haplotypes from the present study are noted as 'Bal' (Suppl. material 1). Haplotypes, identical with ones published previously, are noted with the '=' symbol.

**Figure 4.**
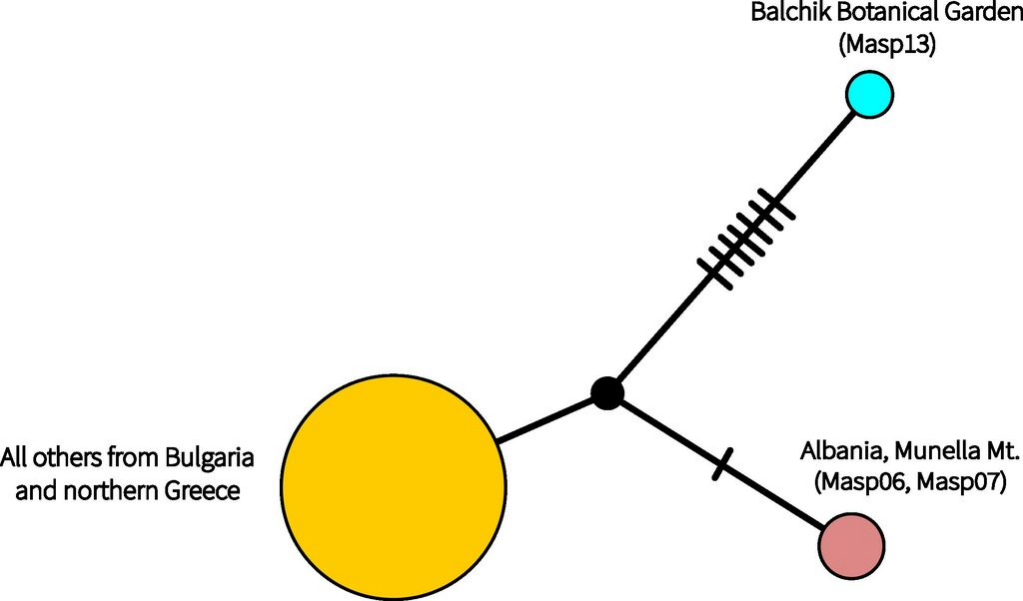
TCS network of selected ITS2 gene sequence of Bulgarian, northern Greece and Albanian samples. Codes correspond to voucher specimens’ number (Suppl. material 1).

The final length of the COI fragment used for phylogeny was 756 bp, from which 166 variable and 76 parsimony informative sites were obtained. The BI tree obtained in this study does not significantly contradict the previously-published phylogenies of Solano et al. (2013) and confirms the main lineages. The newly-sampled Strandzha populations arrange within *M.asper* s.l., forming a sister clade to L3. The majority of the new samples are arranged within L3 (Suppl. material 2). The final length of the ITS2 alignment was 388 bp (including 26 gaps) with 43 variable and 11 parsimony informative sites. The Bayesian ITS2 tree provided strong support for deeper nodes. The majority of the Bulgarian samples arranged together with samples from Greece, Serbia, Slovenia and Croatia. The samples from Strandzha Mts. and the Black Sea coast (Albena) were grouped together with samples from Turkey provided by Solano et al. (2013). The samples from the Balchik Botanical Garden and from Giresun (Turkey) formed a fully supported clade, but its position remained unresolved (Suppl. material 3). The TCS haplotype network of all samples of mitochondrial COI haplotypes confirmed previous findings (Solano et al. 2013, Gojković et al. 2022). The L3 lineage shows a clear star-like pattern with genetic distances within 1–5 bp, while the other lineages show greater diversity and more complex patterns (Fig. 3). All statistics calculated for the L3 lineage (including our own and published data) were negative (Table 1). The nucleotide diversity of the Bulgarian sample of the mitochondrial COI gene sequences demonstrated a relatively high value (pi = 0.018), close to those of Serbia and Montenegro samples. The number of COI haplotypes detected was also relatively high (20).The nuclear TCS network was based on a 5.8S-ITS2 fragment of 373 bp which consisted of selected specimens from Bulgaria, Albania and the northern part of Greece (Fig. 4). Differences from the main cluster were observed in the Albanian samples. Morphologically identified *M.verecundusbulgaricus* (Balchik Botanical Garden) demonstrated a distinct ITS2 genotype for the Balkans, yet similar to a sequence from Giresun, Turkey, published earlier (Solano et al. 2013) (Suppl. material 3).

## Discussion

***Morimusasper* s.l. lineages in the eastern Balkans**

Our study extends the knowledge on the distribution of mitochondrial lineages of *M.asper* in the eastern Balkans, covering most of the territory of Bulgaria, a part of northern Greece and northern Albania (Munella Mt.). New data demonstrates the presence of four mitochondrial lineages of *Morimusasper* s.l. in Bulgaria. Three of these were already defined by Solano et al. (2013) as Lb – HgA; L2 and L3 and one is new – the Str lineage from the Strandzha Mountains (south-eastern Bulgaria, shared partly with the European part of Turkey). The most abundant COI lineage in Bulgaria and the sampled region of northern Greece is L3, confirming the results of Gojković et al. (2022). Haplotypes previously reported from Montenegro, Croatia, Slovenia and Serbia were also found in Bulgaria. The most abundant Bulgarian haplotype (Bal05) was observed in samples from the Danubian Plain, Predbalkan, Shumen Plateau, central and eastern Stara Planina Mts., Rhodope Mts., Pirin Mts., Kresna Gorge and Belasitsa Mts. in Bulgaria, as well as Falakro Mts., Menoikio Mt. and Pangaion Mt. in northern Greece. This haplotype was previously published from Croatia – Velebit Mts. (Solano et al. 2013) and Serbia – Cer Mt. (Gojković et al. 2022). The L3 lineage dominates in most of Bulgaria, but was not found in the easternmost parts of the country close to the Black Sea coast, where L2 is found (Fig. 2). Despite the extensive sampling, only three individuals from L2 were reported, two of them near the Black Sea coast and one, surprisingly, in south-western Bulgaria (near Melnik Town). The Lb lineage was found in the north-westernmost part of the country in the foothills of the Stara Planina Mts. close to the Serbian border. One of the haplotypes (Gorni Lom vill.) was identical to one from Montenegro. This lineage is also found in Croatia, Montenegro, Albania and Serbia (Solano et al. 2013, Gojković et al. 2022 and this study). Bulgaria seems to be the eastern limit of Lb/HgA distribution.

The sample from the type locality of *M.verecundusbulgaricus* belongs to the L2 COI lineage and is identical with a haplotype already reported from Serbia by Gojković et al. (2022). Its nuclear ITS2 gene sequence differs from all Balkan samples (Fig. 4) and is similar to a sequence from the Black Sea region of north-eastern Turkey (Mor ITS2 (5a), reported in Solano et al. (2013)), where *M.verecundusverecundus* Faldermann, 1836 is known to occur (Özdikmen 2022) (Suppl. material 3). This locality, ‘Giresun Dağlari, 3 km N of Kümbet,’ was not represented with mitochondrial DNA in the analyses of Solano et al. (2013). While nuclear data for *Morimus* remain insufficient, some of the most divergent populations are reported along the Black Sea coast (Black Sea region of Turkey, Bulgarian Black Sea coast, Strandzha Mts.). With the exception of the Str lineage in the Strandzha Mts., these populations share similar ITS2 genotypes and L2 mitochondrial DNA, although it remains unclear whether the populations are isolated and associated with morphologically identified *M.verecundus*.

The three individuals found in Strandzha Mts. have three mitochondrial haplotypes, similar to each other, but equally distant from the rest. These formed a clade sister to L3 on the COI phylogenetic tree (Suppl. material 2) though with low support. All specimens from Strandzha were identified as *M.orientalis*, based on morphology, but are genetically distant from individuals studied by Solano et al. (2013) from Turkey, where *M.orientalis* was described for the first time (Reitter 1894). However, all three individuals show the Mor ITS2 (1a) nuclear genotype, which is predominantly found in the eastern Balkans. Due to these relatively high distances in mitochondrial DNA and the unclear taxonomic status of the populations, we currently consider these as a new lineage of *M.asper* s.l. (Str lineage hereafter).

Our results, as well the previous study by Solano et al. (2013), showed intense exchange gene flow between the different populations and genetic lineages of *Morimus* sp. and it is very likely that both *M.verecundus* and *M.orientalis* are subspecies of *M.asper* as Solano et al. (2013) stated, but still, a taxonomic decision could not be made. Similar morphological variability, hybridisation and species delimitation problems have been observed in other Cerambycidae groups, such as the flightless Dorcadionini (Dascălu et al. 2022, Karpiński et al. 2023), as well as in some winged Cerambycini, such as the *Ropalopusungaricus*/*insubricus* group (Karpiński et al. 2020). In all cases, phylogenetic trees, based solely on COI sequences, do not provide delimitation of all species. Adding the ITS2 molecular marker in the case of European and Turkish *Morimus* representatives showed discrepancies in the obtained phylogenetic trees. These contradictions, between COI and ITS2-based phylogenetic trees in grouping of *M.orientalis* samples from Bulgaria and Turkey, as well as of *M.verecundusbulgaricus* and *M.verecundusverecundus* from Turkey and Iran, showed that there is still a gap in our knowledge of the phylogenetic relationships in the genus *Morimus*. The observed discrepancies in the phylogenetic trees of *Morimus* follow a geographical pattern, which, according to Toews and Brelsford (2012), shows differentiation in isolation followed by secondary contact and hybridisation, an indication of a recent speciation. However, to fully understand the phylogenetic relations and species boundaries in *Morimus* in Europe and Turkey, an integrative approach has to be applied by adding more nuclear markers and in-depth morphological analyses, as well as further extensive sampling in Turkey and the Caucasus.

**Dispersal routes**

This study does not provide sufficient data for testing hypotheses about population structure, gene flow or confidently locating glacial refugia of *M.asper*. However, extensive sampling in the eastern Balkans (Bulgaria and northern Greece) bridges a significant knowledge gap regarding the distribution of genetic lineages, reaching the northern Black Sea coast to the east, thus highlighting some dispersal routes. In Bulgaria, the L3 lineage clearly dominates (Fig. 2) showing a typical star-like haplotype network (Fig. 3). Negative values for all test statistics were obtained using Bulgarian samples of L3 (Table 1), though with non-significant p values – possibly because of the low sample size. However, a statistically significant negative Tajima’s D was obtained with all available sequences from L3 (Solano et al. 2013, Gojković et al. 2022, this study) which strongly supports the recent expansion of the lineage. The majority of samples reported from Serbia also belong to the L3 lineage (Gojković et al. 2022), but Lb-HgA is much more common there compared to Bulgaria, suggesting that the main dispersal route of L3 is in the eastern direction.

Prior to this study, the highest diversity of the L2 lineage was mainly reported from Turkey, with isolated haplotypes in Croatia (Solano et al. 2013) and Serbia (Gojković et al. 2022). New data highlight two dispersal routes of this mitochondrial lineage on the Balkan Peninsula: 1) to the north along the Black Sea coast and 2) in a somewhat south-western direction where only separate disconnected populations remain (Melnik, Bulgaria; Sopotnica, Serbia; Biokovo Mountains, Croatia). A separate colonisation event in Europe from the Orient was also suggested by Solano et al. (2013). Although our study does not imply divergence times estimations, one plausible scenario, based on the phylogenetic position and the distribution of L2, is that the L2 lineage colonised the Balkans first and was later largely outcompeted or assimilated by L3 during a later stage of interglacial radiation. Such processes reflect glacial-interglacial cycles and are well-studied (e.g. Taberlet and Cheddadi (2002), Hewitt (2004), Hewitt (2011)). Additionally, levels of genetic exchange between the mitochondrially defined lineages are probably high. Two sampled individuals from south-western Bulgaria, namely Melnik Town and Kalimantsi vill., at ca. 10 km distance, belong to the L2 and L3 COI lineages, respectively, while showing identical ITS2. Similarly, genetically distant mitochondrial haplotypes (Lb/HgA and L3) with identical ITS2 were found close to each other in Albania (Munella Mt.).

All this is in line with a previously discussed scenario based on the association of *Morimus* with beech forests (Romero-Samper and Bahillo 1993) and a post-glacial dispersal out of refugia linked with that of *Fagussylvatica* (Solano et al. 2013). Paleobotanical and genetic data suggest that glacial refugia of *Fagus* in Europe were not restricted to particular areas, but represented small, scattered patches in multiple suitable regions in Europe, from which beech populations rapidly spread as the glacial period ended (Magri 2007). It is reasonable to assume that *Morimus* populations survived glacial periods, restricted to suitable forest habitats and, after climatic conditions improved and suitable habitats spread, lineages started recolonising new areas. In the case of L3, the low nucleotide diversity and highly negative Tajima’s D and other tests reflect this pattern.

The newly-proposed Str lineage is more intriguing, as it is sister to L3, according to our phylogeny (Suppl. material 2) and there is ca. 2.5% distance between them. None of the previously reported lineages was found in the Strandzha Mts., while the genetic diversity of Str seems to be comparatively high (Fig. 3). The three distinct COI haplotypes, belonging to Str, have not been reported in previous studies. While robust conclusions cannot be based solely on COI, we accept that the isolation of Str likely occurred before the last glacial-interglacial cycle (compare mutation rates for COI in Papadopoulou et al. (2010), Pons et al. (2010)). Most likely, populations finding refugia close to the Strandzha Mts. were initially isolated during a glacial stage, but after that, there were no suitable conditions for them to colonise vast areas and to reconnect with others. Geographically, the Strandzha Mts. are surrounded by the Thracian Plain and the Black Sea. While at the end of the Last Glacial Period, humidity in western and central Europe increased, drier climate persisted in the Balkans (Wright et al. 2003, Mauri et al. 2015), especially in lowlands (Connor et al. 2013). Besides, pollen analyses show that *Fagus* was amongst the last tree genera to recolonise the Peninsula (Tonkov 2003, Tonkov et al. 2013, Connor et al. 2013), which presumably delayed the expansion of *Morimus* in the Balkans. According to our genetic data, when beech forests finally established in the plains of the eastern Balkans during the late Holocene (Magri et al. 2006), the most successful colonisers of the newly-appeared habitats were members of the L3 lineage of *M.asper*. The L2 and Str remained restricted to areas closer to their glacial refugia. Moreover, *Morimus* has been found in association with various tree species along its range. For instance, on the Iberian Peninsula, *M.asper* has been reported on trees of the genera *Quercus*, *Alnus*, *Populus*, *Cedrus*, *Larix*, *Pinus* etc. (Romero-Samper and Bahillo 1993 and references therein). In Turkey, *M.orientalis* has been reported on *Quercusconferta*, *Castaneasativa*, *Pinusmaritima* and *Abies* sp., while *M.verecundus* has been reported on *Fagusorientalis*, *Quercus* sp., *Piceaorientalis*, *Pinus* sp. (Özdikmen 2022). The Strandzha Mt. lineage was only found in *F.orientalis* and *Quercus* sp. tertiary relict forests with specific undergrowth and microclimate conditions that are very different from the surrounding vegetation communities (Savev et al. 2015) and is genetically distant from all populations sampled so far (Fig. 3). This could be explained by geographical isolation only or could indicate some stage of the formation of an ecotype, following the model described by Karpiński et al. (2021), triggered by divergent adaptations to some ecological variables. However, currently available data are insufficient to estimate the gene flow between Str and the rest of the lineages and to identify any consistent habitat differences across the range of *Morimus*. Karpiński et al. (2021) state that 'ecotypes occur throughout the geographic range of a species in similar ecological niches'. Regarding the genus *Morimus*, its range is huge and there is no consensus on its systematics, with many populations remaining unsampled. Therefore, it remains unclear whether the observed populations should be considered species, subspecies or ecotypes.

**Conservation considerations**

The taxonomic status of saproxylic beetles of the genus *Morimus* in Europe remains unclear and is highly debated with regard to their conservation and the inclusion of individual taxa in the Habitats Directive (Solano et al. 2013, Gojković et al. 2022). Despite the taxonomic uncertainty, strict protection has to be applied for all lineages of *Morimus* in Bulgaria, although under the taxon *M.funereus*, special attention regarding the rare and localised Lb/HgA, L2 and Str lineages has to be paid when making a management plan for the *M.asper* s.l. in Bulgaria in order to retain the genetic diversity and uniqueness of the Bulgarian populations. The localities of the Lb/HgA lineage fall within the Natura 2000 site BG0001040 West Stara Planina and Predbalkan, where the beech forests are still intact and well preserved. On the other hand, the representatives of the L2 COI lineage are particularly threatened due to the increasing decline and severe fragmentation of the lowland and riparian forests along the Black Sea coast, even if parts of them are protected in Natura 2000 sites and nature reserves. Suitable habitat fragments are separated by large distances (Kostova et al. 2023), preventing gene exchange between the *Morimus* populations there. The deciduous forests around the town of Melnik in the foothills of the Pirin Mountains, where the isolated L2 lineage also was found, are protected as a Natura 2000 site – BG0001028 Middle Pirin – Alibotush. This area requires additional research effort and precautionary measures for the conservation of habitats, as it is the only inland location of the L2 lineage in Bulgaria and indicates the presence of a nearby Pleistocene refugium, probably also for other organisms. The Strandzha Mountains, whose forests descend to the coast of the Black Sea, are of exceptional importance for the genetic diversity of *Morimusasper* s.l., preserving the unique Str lineage of the genus. Although most Strandzha territory is a protected area as a nature park, including five nature reserves and it is a NATURA 2000 site (BG0001007) for its unique habitats, investment plans and logging activities threaten to fragment the deciduous forests. There is an urgent need to adopt a management plan for the Strandzha Nature Park to ensure the protection and continuity of suitable habitats for the Str lineage of *Morimus*. Joint efforts should also be made with the Turkish Forestry Administration in order to conserve this unique genetic lineage.

## Supplementary Material

41DD2A1F-E00B-5E07-866F-683C0C9FAF55 10.3897/BDJ.12.e116619.suppl1 Supplementary material 1 Detailed sample information Data type Table Brief description Contains information about voucher specimens, localities, coordinates, COI and ITS2 accession numbers at GenBank, COI haplotypes (obtained from this study are noted as “Bal”), COI lineages and nuclear ITS2 comparison with previous studies (Solano et al. 2013; Gojković et al. 2022). File: oo_963111.xlsx https://binary.pensoft.net/file/963111 Kostova R, Borissov S, Bobeva A, Bekchiev R

E5F9CCAE-9AF1-56B3-A010-2DD4FB040E23 10.3897/BDJ.12.e116619.suppl2 Supplementary material 2 Bayesian inference tree using all available COI sequences Data type phylogenetic tree Brief description Bayesian inference tree using all available COI sequences (Solano et al. 2013, Gojković et al. 2022 and present data) File: oo_943095.pdf https://binary.pensoft.net/file/943095 Kostova R, Borissov S, Bobeva A, Bekchiev R

BD618B22-3425-50A5-9F8C-3B369AB3491D 10.3897/BDJ.12.e116619.suppl3 Supplementary material 3 Bayesian inference tree using all available ITS2 sequences Data type phylogenetic tree Brief description Bayesian inference tree using all available ITS2 sequences (Solano et al. 2013, Gojković et al. 2022 and present data) File: oo_962934.pdf https://binary.pensoft.net/file/962934 Kostova R., Borisov S., Bobeva A., Bekchiev R.

## Figures and Tables

**Table 1.** Nucleotide diversity and neutrality tests for L2 and L3 COI lineages including our own and published data (Solano et al. 2013, Gojković et al. 2022, present study). The significant value is marked with an asterisk.

Lineage	Samples	Haplotypes	S	Nucleotide diversity	Tajima's D	Significance of Tajima's D (p)	CI (99%) of D H_0_: SNM	D'	F'	Fu’s Fs
L3	68	29	31	0.00389	-2.3355*	< 0.001	-1.955÷2.677	-3.4095	-3.6073	-45.984
L2	11	9	14	0.00551	-0.91732	> 0.10	-1.889÷2.032	-1.1067	-1.1877	-5.556
L3 Bulgaria	45	12	13	0.00351	-1.62679	> 0.10	-2.043÷2.329	-1.9921	-2.1570	-12.165
Bulgaria all lineages	54	20	49	0.01891	-0.09968	> 0.10	-2.048÷2.144	0.3837	0.2783	-9.570
