# Supplementary figures and images for: At a crossroads: Genetic lineages and dispersal routes of Morimusasper (Sulzer, 1776) s.l. (Coleoptera, Cerambycidae) in Bulgaria
Source: Biodivers Data J. 2024 Feb 5;12:e116619. doi: 10.3897/BDJ.12.e116619 (PMC10862347; doi:10.3897/BDJ.12.e116619)

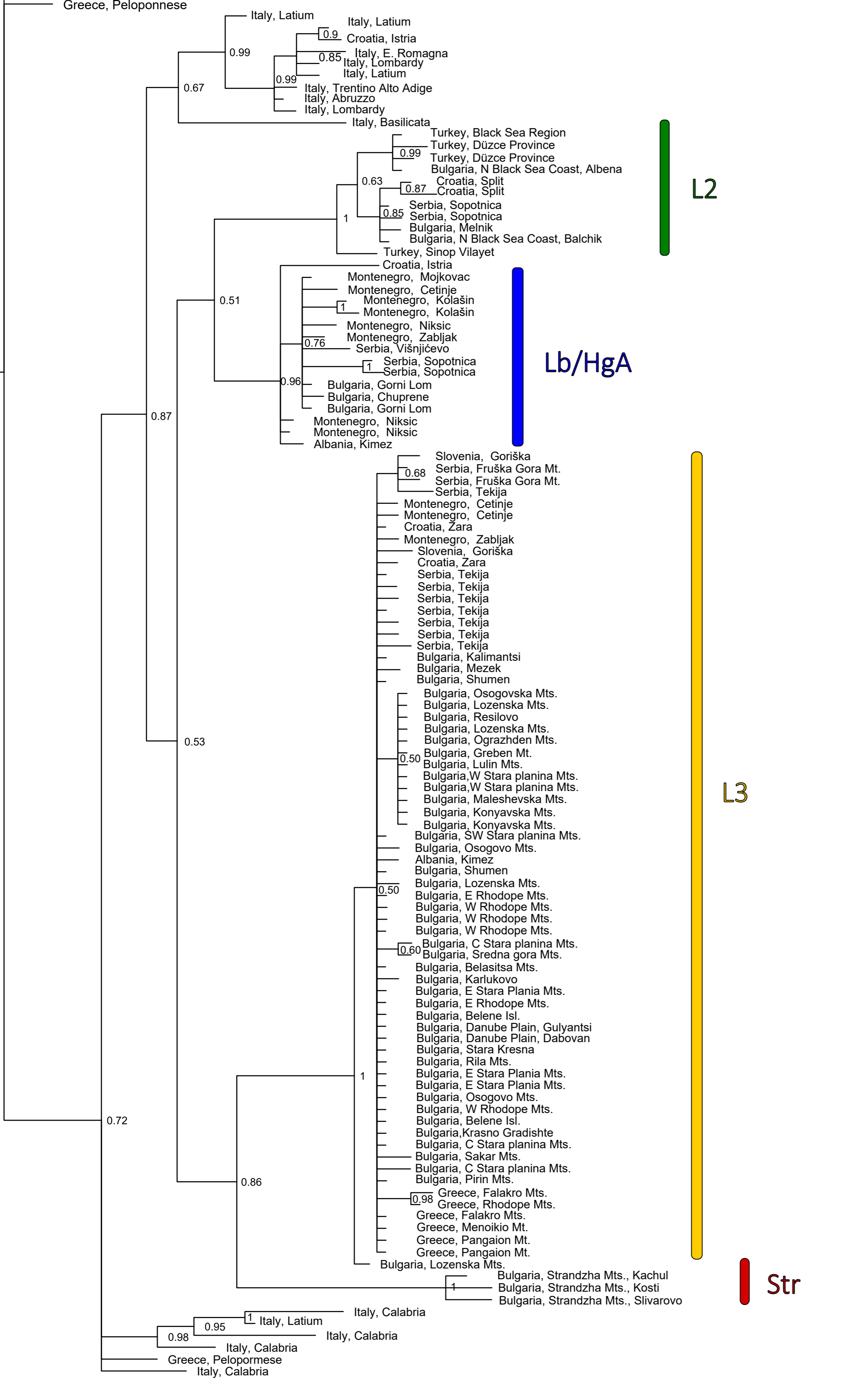

Supplement: Supplementary material 2 — Bayesian inference tree using all available COI sequences [file bdj-12-e116619-s002.pdf]

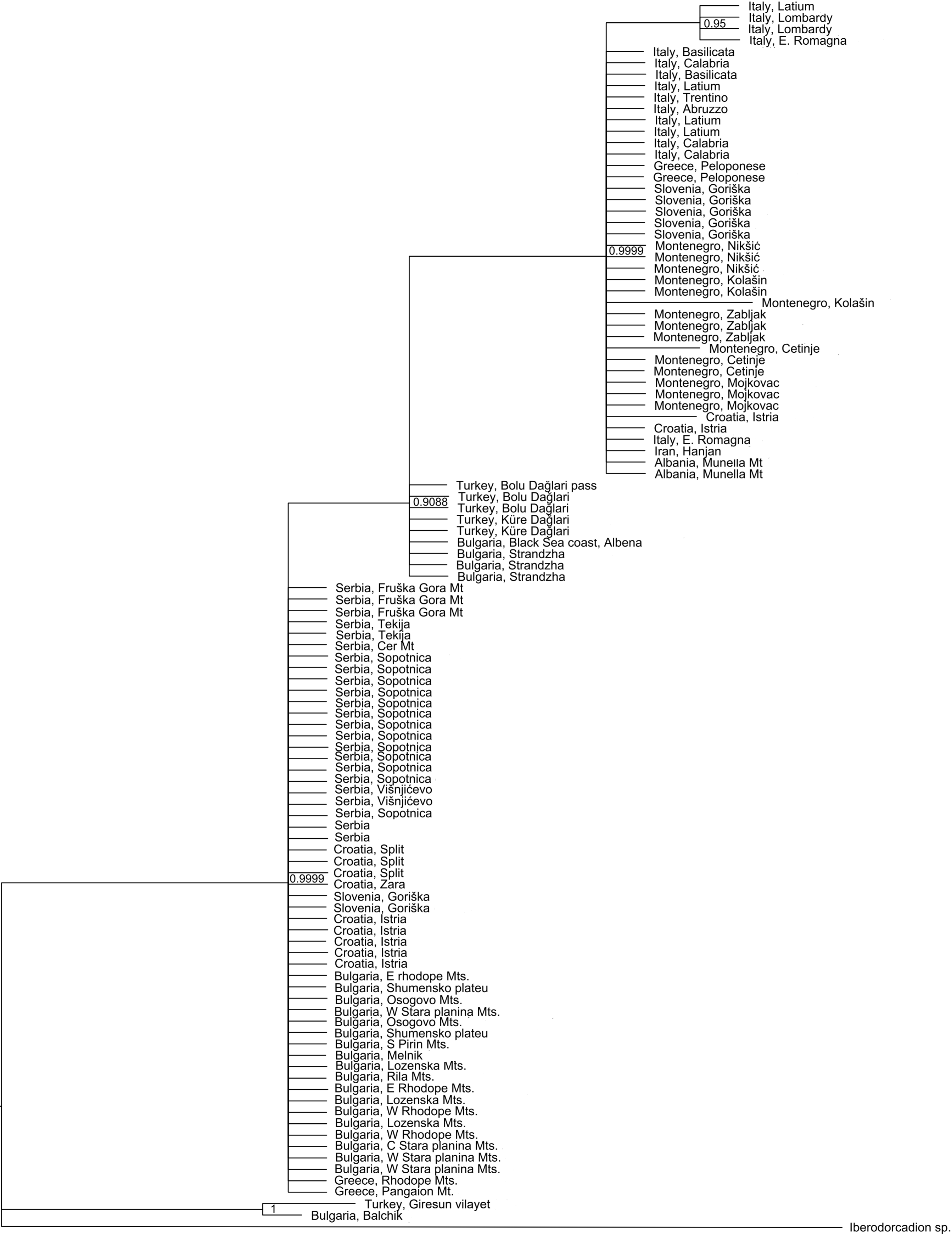

Supplement: Supplementary material 3 — Bayesian inference tree using all available ITS2 sequences [file bdj-12-e116619-s003.pdf]
